# Impact of a single round of mass drug administration with azithromycin on active trachoma and ocular *Chlamydia trachomatis* prevalence and circulating strains in The Gambia and Senegal

**DOI:** 10.1186/s13071-019-3743-x

**Published:** 2019-10-22

**Authors:** Emma M. Harding-Esch, Martin J. Holland, Jean-François Schémann, Ansumana Sillah, Boubacar Sarr, Linus Christerson, Harry Pickering, Sandra Molina-Gonzalez, Isatou Sarr, Aura A. Andreasen, David Jeffries, Chris Grundy, David C. W. Mabey, Bjorn Herrmann, Robin L. Bailey

**Affiliations:** 1 London School of Hygiene & Tropical Medicine, Keppel Street, London, WC1E 7HT UK; 2 Medical Research Council Laboratories, PO Box 273, Fajara, Banjul, The Gambia; 3 Institut de Recherche pour le Développement (IRD), Dakar, Sénégal; 4 National Eye Health Programme, Ministry of Health and Social Welfare, Kanifing, The Gambia; 5 Programme National de Lutte Contre la Cécité, Ministère de la Santé, BP 3817, Dakar, Sénégal; 6 Department of Clinical Microbiology, Uppsala University, Uppsala, Sweden

**Keywords:** Active trachoma, *Chlamydia trachomatis*, Ocular, Mass drug administration, Azithromycin, Prevalence, Whole-genome sequence, Organism load, *ompA*, Multi locus sequence typing

## Abstract

**Background:**

Mass drug administration (MDA) with azithromycin is a cornerstone of the trachoma elimination strategy. Although the global prevalence of active trachoma has declined considerably, prevalence persists or even increases in some communities and districts. To increase understanding of MDA impact, we investigated the prevalence of active trachoma and ocular *C. trachomatis* prevalence, organism load, and circulating strains at baseline and one-year post-MDA in The Gambia and Senegal.

**Methods:**

Pre- and one-year post-MDA, children aged 0–9 years were examined for clinical signs of trachoma in six Gambian and 12 Senegalese villages. Ocular swabs from each child’s right conjunctiva were tested for evidence of ocular *C. trachomatis* infection and organism load (*ompA* copy number), and *ompA* and multi-locus sequence typing (MLST) was performed.

**Results:**

A total of 1171 children were examined at baseline and follow-up in The Gambia. Active trachoma prevalence decreased from 23.9% to 17.7%, whereas ocular *C. trachomatis* prevalence increased from 3.0% to 3.8%. In Senegal, 1613 and 1771 children were examined at baseline and follow-up, respectively. Active trachoma prevalence decreased from 14.9% to 8.0%, whereas ocular *C. trachomatis* prevalence increased from 1.8% to 3.6%. Higher organism load was associated with having active trachoma and severe inflammation. Sequence typing demonstrated that all Senegalese samples were genovar A, whereas Gambian samples were a mix of genovars A and B. MLST provided evidence of clustering at village and household levels and demonstrated differences of strain variant frequencies in Senegal, indicative of an “outbreak”. MLST, including partial *ompA* typing, provided greater discriminatory power than complete *ompA* typing.

**Conclusions:**

We found that one round of MDA led to an overall decline in active trachoma prevalence but no impact on ocular *C. trachomatis* infection, with heterogeneity observed between villages studied. This could not be explained by MDA coverage or number of different circulating strains pre- and post-MDA. The poor correlation between active trachoma and infection prevalence supports the need for further work on alternative indicators to clinical signs for diagnosing ocular *C. trachomatis* infection. MLST typing has potential molecular epidemiology utility, including better understanding of transmission dynamics, although relationship to whole-genome sequence variability requires further exploration.

## Background

Trachoma is the leading infectious cause of blindness and targeted for elimination as a public health problem by 2020 [1]. Initial infection presents clinically as active trachoma [trachomatous inflammation-follicular (TF) and/or trachomatous inflammation-intense (TI)]. Years of reinfection can lead to scarring of the conjunctiva, causing contraction of the eyelid and eyelashes to turn inwards to scratch against the eyeball (trichiasis), which can result in corneal opacity and blindness. Mass drug administration (MDA) with antibiotics is one component of the World Health Organization (WHO)-endorsed SAFE strategy for trachoma elimination: surgery for trichiasis; antibiotics to clear ocular *Chlamydia trachomatis* infection; and facial cleanliness and environmental improvement to reduce infection transmission [2]. WHO recommends MDA, as well as “F” and “E”, at the district level (which for trachoma elimination purposes WHO defines as “the normal administrative unit for health care management consisting of a population unit between 100,000–250,000 persons”) for five years where TF prevalence is ≥ 30% in 1–9 year-olds, three years where TF is 10.0–29.9%, and one year where TF is 5.0–9.9%, before reassessment of TF prevalence [3].

Since 1999, the single oral dose antibiotic, azithromycin, has been donated by its manufacturer, Pfizer, for use in SAFE programmes for distribution *via* the International Trachoma Initiative (ITI). The Gambia and Senegal lie in the dry, arid, Sahel belt of West Africa. In The Gambia, evidence from two national surveys carried out in 1986 and 1996 showed a 54% reduction in the prevalence of active trachoma in 0–14 year-olds from 10.4% to 4.9% [4]. Subsequent survey data demonstrated 0.3% ocular *C. trachomatis* infection but greater than 10% TF prevalence [5], suggesting The Gambia was on course for trachoma elimination. However, variation in prevalences between communities has also been demonstrated, including as a result of re-introduction of infection from untreated communities in Senegal [6, 7], which could potentially hinder the success of elimination programmes. For Senegal, a national trachoma survey conducted in 2000 estimated that the prevalence of active trachoma in children aged under 10 years and not living near Dakar was 10.8% [8]. A 2004 study in the Nioro Department (within Kaolack Region, which had a prevalence of 6.8% in the 2000 survey), had an active trachoma prevalence of 17.4% in children aged 2–5 years [9]. These data similarly demonstrated heterogeneity in active trachoma prevalence throughout the country.

Randomised controlled trials demonstrate that MDA with azithromycin reduces the prevalence of active trachoma and ocular *C. trachomatis* infection in communities and districts [10, 11]. To date, eight countries have been validated as having eliminated trachoma as a public health problem by WHO since 2012, and it is projected that 70% of formerly endemic districts will have met the 5% TF elimination threshold by 2020 [12]. However, studies (mainly conducted in medium-high prevalence settings) have demonstrated heterogeneous impact of MDA, which has potential implications for the success of the global effort towards trachoma elimination as a public health problem, in reaching and subsequently maintaining TF prevalence below the elimination threshold. Some communities experience elimination of active trachoma and/or infection, some observe prevalence decreases but not to the 5% elimination TF threshold, and others encounter rapid re-emergence [13–20]. Explanations for these findings include baseline prevalence of active trachoma and/or ocular *C. trachomatis* infection, treatment coverage, re-infection from untreated communities, random fluctuation, seasonal effects, secular trend, and regression to the mean. An additional potential explanation is strain diversity. In The Gambia, the number of strains (as determined by *ompA* sequence variation) reduced after MDA, with one strain associated with higher organism load [21]. The data did not support the hypothesis that *ompA* polymorphisms were maintained within the population by immune selection pressure, and it was suggested that typing systems including other polymorphic loci could, through greater discrimination, help better define ocular *C. trachomatis* infection population dynamics and the impact of MDA. The multi-locus sequence typing (MLST) system developed by Klint et al. [22] scheme does not exclusively use either short amplicons or housekeeping genes, and provides greater discrimination than *ompA* alone for genital *C. trachomatis* infection typing [23, 24].

This study, conducted as part of a study evaluating a prototype point-of-care test (POCT) for ocular *C. trachomatis* infection [25], took place before The Gambia had received its azithromycin donation *via* the ITI, whereas MDA of districts in Senegal commenced in 2005. To understand the reasons for variation in MDA impact in these two low prevalence countries at different programmatic stages, and since MDA is distributed annually, we aimed to investigate the one-year impact of a single MDA with azithromycin on active trachoma and ocular *C. trachomatis* prevalence, organism load, circulating strains and spatial distribution in The Gambia (villages treated in the absence of district-level MDA) and Senegal (villages treated as part of MDA of the whole district). As a sub-study, we also aimed to evaluate the relationship between MLST and whole-genome sequence (WGS) variation using a population from Bijagos Islands, Guinea-Bissau.

## Methods

### Field data collection

In The Gambia, six villages in the North Bank and Lower River Regions were selected based on previous survey data [5] and identified by Community Ophthalmic Nurses as having a village prevalence of TF ≥ 10% in 1–9 year-old children. These villages met the criteria for MDA in 2006 [26] prior to The Gambia receiving its azithromycin donation in 2007. In Senegal, the sanitary district of Bambey within the medical region of Diourbel, which had the highest trachoma prevalence in the 2000 national survey [8], was identified by the Senegalese National Eye Care Programme (NECP) as requiring MDA. Prior to MDA, twelve geographically dispersed villages under the health post of Keur Samba Kane were selected for the study.

Field data collection methods have been described in detail elsewhere [25]. Fieldwork in The Gambia took place in March–May 2006 (baseline) and June–July 2007 (one-year follow-up). In Senegal, the baseline study was in January–February 2007 and the one-year follow-up was in March–May 2008.

At both time-points (baseline and one-year follow-up), a census of the *de facto* population was made of the selected villages, and global positioning system (GPS) coordinates collected for every household using an eTrex® H handheld device (Garmin (Europe) Ltd., Southampton, UK). In both settings, a household was defined as individuals who shared a common cooking pot. After written (signature or thumbprint) informed consent by their guardians, all children aged 0–9 years were examined for clinical signs of trachoma by experienced observers using the WHO simplified grading system [27]. All the grading was performed by one ophthalmic nurse in The Gambia, and another in Senegal. The graders were validated using a WHO slide pack and were required to achieve a chance corrected agreement (Cohen’s kappa statistic [28]) of ≥ 0.8 for TF, TI and TS (trachomatous scarring). Two Dacron swabs (Quelab Laboratories, Montreal, Canada) were then taken from each child’s right upper conjunctiva using a standardised technique [29].

### Mass drug administration with azithromycin

After examination at baseline, the communities were offered MDA with azithromycin. Individuals aged 14 years and above were given the recommended 1 g of azithromycin; children received treatment on the basis of 20 mg/kg, using height as a surrogate for weight, up to 1 g. Where azithromycin was contra-indicated (children aged under 6 months and pregnant women), two vials of 1% tetracycline eye ointment were given, with instructions on how to apply it. In The Gambia, MDA was distributed by the research team in the six study villages. Individuals who presented themselves who were not previously registered on the enumeration form were added if they were permanent residents in the household, and all were treated. The reason for absence was noted for any recorded individual not present during the treatment distribution. In Senegal, treatment of the Bambey district was distributed by the Senegalese NECP. Despite instructions to the MDA team that a census of household members be made before treatment was distributed (the study census not being used as MDA was not distributed by the research team), only individuals who were given treatment were recorded in notebooks. Names from the NECP notebooks were matched with the study census to calculate treatment coverage.

### Detection and copy number estimation of *C. trachomatis* infection

Samples, kept on ice in the field, were transferred to a − 20 °C freezer within 10 hours. *Chlamydia trachomatis* infection was detected in stored samples using the Amplicor *Chlamydia trachomatis/Neisseria gonorrhoeae* (CT/NG) Polymerase Chain Reaction (PCR) assay (Roche Molecular Systems, Indianapolis, IN, USA) according to the manufacturer’s instructions, except that a previously published method was used for sample extraction [29]. At baseline, the first-collected swab was processed by a POCT [25] and the second-collected swab was processed by Amplicor. In the one-year follow-up, the first-collected swab was processed by Amplicor and the second-collected swab was archived. All samples were processed within 6 months of collection.

Amplicor-positive samples were purified using the QIAamp DNA Minikit 250 (Qiagen, Crawley, UK). Two 4 µl replicate samples were each processed in two real-time quantitative PCR assays which used a total of three primers. The first assay used a genovar A-specific forward primer and a common reverse primer as previously described [29]. The second assay used the genovar B-specific forward primer (5′-TCT gTT gTT gAg TTg TAT ACA gAT AC-3′) (Sigma-Genosys, Gillingham, UK) with the same common reverse primer.

Gambian sample *ompA* copy number estimates were performed on a LightCycler (Roche Diagnostics, Indianapolis, IN, USA). For both genovars, samples were denatured at 95 °C for 15 min. The genovar A samples were then subjected to 45 cycles of thermal cycling at 95 °C for 15 s, 59 °C for 20 s, 72 °C for 15 s, 79 °C for 5 s. The genovar B cycling conditions were 45 cycles of 94 °C for 15 s, 55 °C for 30 s, 72 °C for 30 s, 79 °C for 5 seconds. The Senegalese follow-up samples were processed on a Rotor-Gene RG3000 (Qiagen, Crawley, UK) with the same cycling conditions. Samples that did not amplify were diluted 1:5, and 1:10 if necessary. The number of *ompA* copies per swab, to represent estimated organism load, was estimated using a previously described method [29].

### Sequence typing

MLST was performed using the system developed by Klint et al. [22] based on the determination of sequences at the five loci *hctB*, CT058, CT144, CT172 and *pbpB*. *OmpA* and MLST sequencing were attempted on all Amplicor-positive Gambian baseline samples, but subsequent attempts were limited to samples estimated to contain more than 30 *ompA* copies/swab. PCR amplification and sequencing of the MLST target regions were performed on the Amplicor extract as previously reported [23] at the Uppsala University, Sweden. Sequence types were assigned profiles using the nomenclature from the hr-CT-MLST dataset [30]. Minimum-spanning trees were constructed using BioNumerics 7.6 created by Applied Maths NV (http://www.applied-maths.com).

*OmpA* amplification and sequencing took place at the London School of Hygiene & Tropical Medicine (LSHTM), UK. One microliter of the QIAamp purified extract was first amplified in a reaction mixture containing 12.5 µl HotStarTaq master mix (Qiagen, Crawley, UK), 4.5 µl of DEPC-treated sterile water, and 1 µl of each primer at 12.5 µM. The forward primer (118F: 5′-ATT gCT ACA ggA CAT CTT gTC-3′) and reverse primer (1163R: 5′-Cgg AAT TgT gCA TTT ACg TgA g-3′) (Sigma-Genosys, Gillingham, UK), generated an amplicon of approximately 1.28 kb of the *ompA* gene. The reaction mixture was amplified using a touch-down PCR with the following conditions: 95 °C for 15 min; 5 cycles of 94 °C for 10 s, 63 °C for 30 s and 72 °C for 1 min; 35 cycles of 94 °C for 10 s, 60 °C for 30 s and 72 °C for 1 min; 72 °C for 12 min; 4 °C for 30 s; followed by holding at 15 °C.

Sequencing of *ompA* was performed using primers 118F and 1163R and inner primers CT2F (5′-TCC AAT ATg CTC AAT CTA AAC CTA AA-3′) and CT2R (5′-TTT Agg TTT AgA TTg AgC ATA TTg gA-3′). Each reaction mix contained 2.9 µl DEPC-treated sterile water, 0.3 µl BigDye^®^ Terminator v3.1 Ready Reaction Mix and 2 µl 5× sequencing buffer (Applied Biosystems, Foster City, CA, USA), 2 µl primer (at 1.5 µM), and 3 µl amplified product. The mixture was amplified in 25 cycles of 96 °C for 10 s, 50 °C for 5 s and 60 °C for 120 s. Amplicons were sequenced on a 3730xl sequencer (Applied Biosystems, Foster City, CA, USA). Sequences were analysed using Seqscape (ABI, Foster City, CA, USA) and 4Peaks (http://mekentosj.com/). Base-calling and assignment of quality values were performed using the ‘KB’ Basecaller (ABI, Foster City, CA, USA). Mixed bases were called when secondary peaks had fluorescence intensity maxima greater than 0.65 times that of the corresponding primary peaks.

Contiguous sequences were aligned against reference sequences from A/HAR 13 (GenBank: NC_007429, genovar A) and B/Jali20/OT (GenBank: NC_012686, genovar B). The resulting sequence alignments were trimmed at the ends until high quality (Q20) base-calls were present at all positions and in all samples. Allele assignments were based upon variation within these ‘clear ranges’, which were 1092 base-pairs for genovar A and 354 base-pairs for genovar B.

Sequencing of *ompA* targeted an extended region compared with the hr-CT-MLST dataset [30], therefore additional discrimination was achieved for some samples otherwise considered to be the same sequence type (ST). STs separated only by extended *ompA* genotyping were assigned the same ST with an additional alphanumeric suffix to indicate discrimination (STs 118d1, 118a1, 118a2, 118d2, 571, 572).

For the sub-study evaluating the resolution achieved between MLST and WGS variation, complete *ompA* typing, WGS and MLST were compared in a population from the Bijagos Islands, Guinea-Bissau. A set of 71 WGS from the Bijagos Islands [31], with inferred MLST and *ompA* types, was utilised to compare these three approaches and their utility in epidemiological studies.

### Statistical analyses

Results were double-entered and verified in Microsoft Access (MS Access v2000/2003XP). Data cleaning and analyses were performed in Stata (v9.2, STATA Corp., College Station, TX, USA). Population descriptive analyses were conducted, and the immigration rate (Number of immigrants (residents only present at follow-up) / Total number of residents at follow-up), and emigration rate (Number of emigrants (residents only present at baseline) / Total number of residents at baseline) were calculated. GPS data were mapped using ArcGIS 9.2 (Environmental Systems Research Institute, Inc. Redlands, CA, USA). Statistical significance was determined at the 5% level. A chi-square test of proportions was used to assess active trachoma (TF and/or TI) and ocular *C. trachomatis* infection prevalence data at baseline and follow-up. Comparison of median organism load between baseline and follow-up was performed using a non-parametric K-sample test on the equality of medians for unmatched data.

MLST analyses are mainly descriptive due to the small number of samples and the large number of variants. Fisher’s exact test was used to look for intra-country differences in variant frequency pre- and post-treatment, and to assess the contribution of the six loci to the resolution of strain differences.

For each sample, individual nucleotide sequences at the six loci were concatenated and aligned using MUSCLE v.3.8 [32]. Genetic (Hamming) distances between these concatenates were calculated and plotted using PhyML [33]. To determine if there was evidence of geographical clustering of similar variants, the Hamming distances between pairs of samples in the same, and in different, cluster strata (village and household) were compared using the Mann-Whitney non-parametric test. Simulation experiments were carried out in R [34] to test whether there was over-representation of short Hamming distances among pairs of geographically related samples, and, further and conversely, whether there was over-representation of short geographical distances between pairs of samples separated by null or short Hamming distances. These experiments were conducted by making 10,000 random redistributions of the MLST combined with *ompA* typed samples to their associated GPS coordinates.

A numerical index of discriminatory ability was estimated using Hunter & Gaston’s application of Simpson’s index of diversity [35]

$$D = 1 - \frac{1}{{N\left( {N - 1} \right)}}\mathop \sum \limits_{j = 1}^S {x_j}\left( {{x_j} - 1} \right)$$

where *N* is the number of unrelated strains tested, *s* the number of different types and *xj* the number of strains belonging to the *j*th type.

D, which takes values between 0 and 1, is the probability that two strains randomly chosen from the sample will be of different types. Typing methods leading to D values of 0.95 or higher are considered highly suitable for molecular epidemiology [36].

To infer MLST and *ompA* types from whole-genome sequences, MLST types were determined from filtered reads using stringMLST [37] and the MLST^6^ (the five MLST regions combined with *ompA*) database [38]. *OmpA* sequences were extracted from filtered reads by aligning to three reference genomes (A/Har13, B/Jali20 and C/TW3) with Bowtie2 [39], variant calls were identified with SAMtools/BCFtools [40]. The *ompA* sequence with the lowest percentage-missing calls per whole-genome sequence was used in downstream analyses.

For phylogenetic analyses, MLST^6^ sequences were concatenated to create a complete MLST sequence per individual. Multiple MLST and genome alignments were generated using progressiveMauve. Phylogenies were computed using RaxML [41] and visualised in R. MLST and WGS phylogenies were compared using R package *dendextend* [42] to determine differences in resolution achieved.

## Results

### Study participation

In The Gambia, 3376 individuals were censused at baseline, of whom 1289 (38.2%) were children aged 0–9 years. At follow-up, 3220 individuals were censused, 1206 (37.5%) of whom were children. Examination was performed on 1171 of these children at both baseline (90.8%) and the one-year follow-up (97.1%). In Senegal, 4822 (1669 children aged 0–9 years, 34.6%) and 4662 (1807 children, 38.8%) individuals were censused at baseline and follow-up, respectively. Of the children, 1613 (96.6%) were examined at baseline and 1771 (98.0%) at follow-up. A summary of the trachoma indicators, methodology, and sample size for each methodology is provided in Table 1.

**Table 1:** Summary of the trachoma indicators, methodology, and sample size

Trachoma indicator	Methodology	Sample size (children aged 0–9 years)				Total
		The Gambia		Senegal		
		Baseline (March–May 2006)	One year follow-up (June–July 2007)	Baseline (January–February 2007)	One year follow-up (March–May 2008)	
Children aged 0–9 years examined for active trachoma (TF and/or TI)	WHO simplified grading system [27]	1171	1171	1613	1771	5726
Ocular *C. trachomatis* infection	Amplicor PCR (Roche Molecular Systems)	1171	1171	1613	1771	5726
Organism load of Amplicor-positives	*ompA* copy number estimates using two real-time quantitative PCR assays for genovars A and B separately [29]	35	45	29	64	173
Multi-locus sequence typing (MLST)	Determination of sequences at the five loci: *hctB*, CT058, CT144, CT172, *pbpB* [22, 23]	13	18	13	29	73
*ompA* sequencing	Sequences aligned against reference sequences from A/HAR 13 (NC_007429, genovar A) and B/Jali20/OT (NC_012686, genovar B), with alignments trimmed at the ends until high quality (Q20) base-calls were present at all positions	19	21	16	38	94
MLST^6^	The five MLST regions combined with *ompA*	14	18	12	28	72
Whole-genome sequencing (WGS)	Set of 71 WGS from the Bijagos Islands with inferred MLST and *ompA* types [40]	na	na	na	na	71

*Abbreviations*: na, not applicable;TF, trachomatous inflammation-follicular; TI, trachomatous inflammation-intense; PCR, polymerase chain reaction

### Treatment coverage

**Fig. 1:**
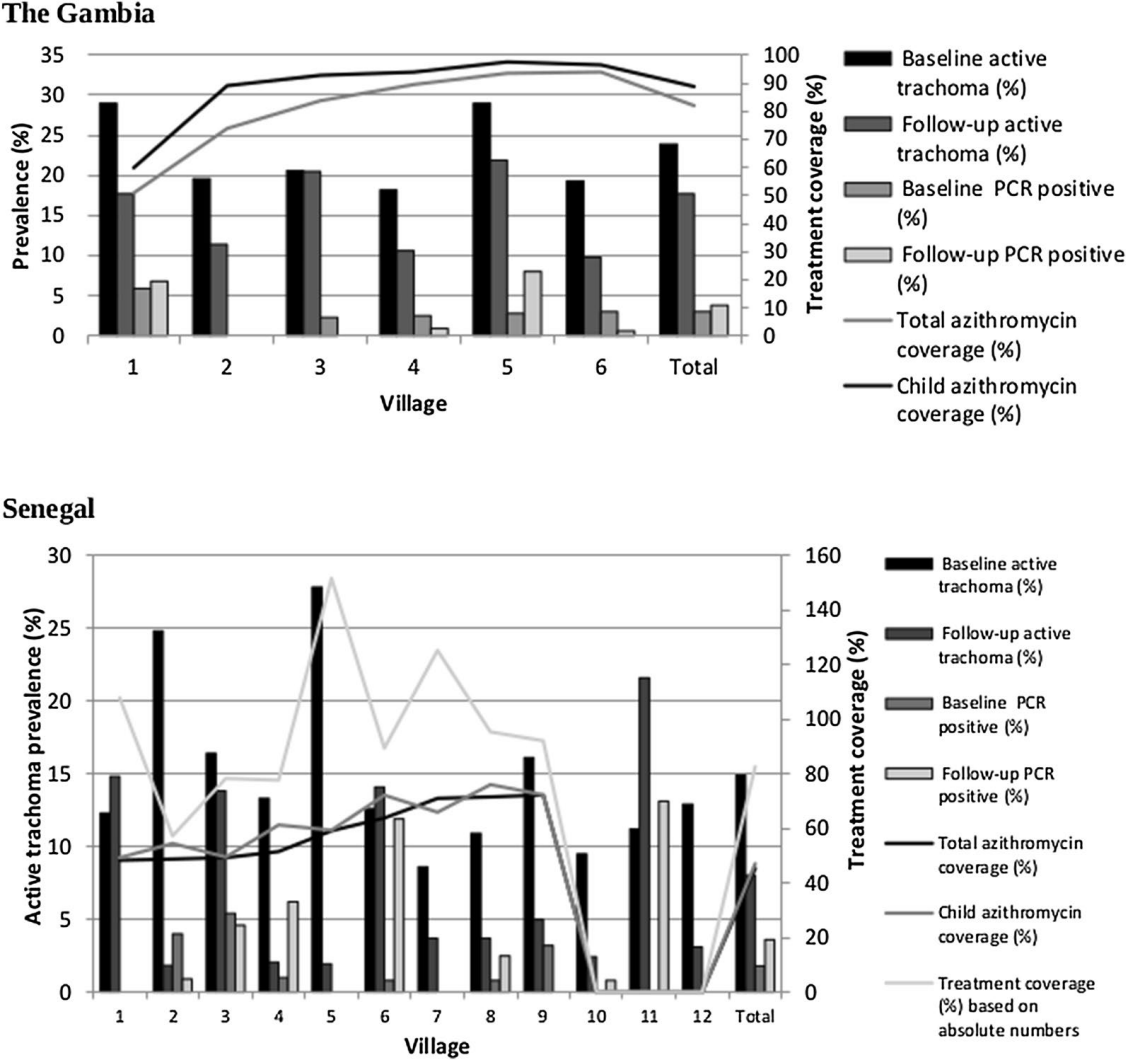
Prevalence of active trachoma (TF and/or TI) and ocular *C. trachomatis* infection in 0–9 year-olds at baseline and follow-up in relation to azithromycin treatment coverage in The Gambia and Senegal

In The Gambia, all 3376 baseline community members were accounted for as either having received azithromycin, tetracycline eye ointment (TEO), or no treatment. Overall treatment coverage with azithromycin was 82.0% (84.1% with azithromycin and TEO). In children aged 0–9 years, the corresponding figures were 88.8% and 88.9% (Fig. 1a). In Senegal, 2444 of the 4822 (50.7%) censused community members were not identified in the NECP treatment record, resulting in an unknown treatment status. This is largely due to no treatment data being available for three of the 12 Senegalese villages. The overall treatment coverage of the censused population was 45.3% with azithromycin (46.2% with azithromycin and TEO). For children aged 0–9 years, the corresponding values were 47.0% and 48.3%. If the absolute number of individuals treated by the NECP in their notebooks is divided by the absolute number of people censused by the study team, overall treatment coverage was 82.6% (Fig. 1b).

### One-year impact of MDA on active trachoma (TF and/or TI) prevalence

In The Gambia, the prevalence of active trachoma in 0–9 year-olds was 23.9% (280/1171; 95% confidence intervals, CI: 21.5–26.5%) at baseline and 17.7% (207/1171; 95% CI: 15.5–20.0%) at follow-up (Table 2, Fig. 1a), representing a 25.9% decrease. For TF in the 1–9 year age group (the indicator and age group used for WHO programmatic decision-making), the overall and individual village TF prevalences continued to surpass the 5% WHO threshold for elimination of trachoma as a public health problem. However, the follow-up TF prevalence (18.8%, 196/1042) was significantly lower than that at baseline (*χ*^2^ = 10.4793, *df* = 1, *P* = 0.001). At the village level, the prevalence of TF fell in all villages except village 3 where it increased marginally from 22.3% to 22.7%.

**Table 2:** Correlation between trachoma clinical signs and ocular *C. trachomatis* infection as detected by Amplicor in children aged 0–9 years

Study	No. of children examined	No. of children with TF (%) [95% CI]^a^	No. of children with TI (%) [95% CI]^a^	No. of infected children (%) [95% CI]^a^	No. of children with TF who were infected (%) [95% CI]^a^	No. of infected children who had TF (%) [95% CI]^a^	No. of children with TI who were infected (%) [95% CI]^a^	No. of infected children who had TI (%) [95% CI]^a^	No. of children with active trachoma who were infected (%) [95% CI]^a^	No. of infected children who had active trachoma (%) [95% CI]^a^
Gambia baseline	1171	280 (23.9) [21.5–26.5]	2 (0.2) [0.02–0.6]^c^	35 (3.0) [2.1–4.]	24/280 (8.6) [5.6–12.5]	24/35 (68.6) [50.7–83.1]	0/2 (0) [0–84.2 ^b^]	0/35 (0) [0–100.0 ^b^]	24/280 (8.6) [5.6–12.5]	24/35 (68.6) [50.7–83.1]
Senegal baseline	1613	216 (13.4) [11.8–15.2]	38 (2.4) [1.7–3.2]^d^	29 (1.8) [1.2–2.6]	24/216 (11.1) [7.3–16.1]	24/29 (82.8) [64.2–94.2]	4/38 (10.5) [2.9–24.8]	4/29 (13.8) [3.9–31.7]	24/240 (10.0) [6.5–14.5]	24/29 (82.8) [64.2–94.2]
Gambia FU	1171	202 (17.3) [15.1–19.5]	8 (0.7) [0.3–1.3]^e^	45 (3.8) [2.8–5.1]	29/202 (14.4) [9.8–20.0]	29/45 (64.4) [48.8–78.1]	3/8 (37.5) [8.5–75.5]	3/45 (6.7) [1.4–18.3]	29/207 (14.0) [9.6–19.5]	29/45 (64.4) [48.8–78.1]
Senegal FU	1771	135 (7.6) [6.4–9.0]	18 (1.0) [0.6–1.6]^f^	64 (3.6) [2.8–4.6]	39/135 (28.9) [21.4–37.3]	39/64 (60.9) [47.9–72.9]	6/18 (33.3) [13.3–59.0]	6/64 (9.4) [3.5–19.3]	39/142 (27.5) [20.3–35.6]	39/64 (60.9) [47.9–72.9]

^a^95% confidence interval

^b^One-sided, 97.5% confidence interval

^c^Both children also had TF

^d^14 of these children also had TF

^e^Three of these children also had TF

^f^11 of these children also had TF

In Senegal, there was a 46.3% decrease in active trachoma prevalence in 0–9 year-olds, from 14.9% (240/1613; 95% CI: 13.2–16.7%) at baseline to 8.0% (142/1771; 95% CI: 6.8–9.4%) at follow-up (Table 2, Fig. 1b). In Senegal, the overall TF prevalence in 1–9 year-olds (8.2%, 133/1619) fell below the 5% threshold, and was significantly lower than at baseline (*χ*^2^ = 26.9905, *df* = 1, *P* < 0.001). Compared with baseline, the prevalence of TF decreased in all villages except in villages 1, 6, and 11. Only 4 villages had a prevalence above 10% at follow-up: villages 1 (15.4%), 3 (14.2%), 6 (15.4%) and 11 (24.6%).

### One-year impact of MDA on ocular *C. trachomatis* infection prevalence

In The Gambia, 3.0% (35/1171; 95% CI: 2.1–4.1%) of children aged 0–9 years-old were Amplicor-positive at baseline compared with 3.8% (45/1171; 95% CI: 2.8–5.1%) at follow-up (Table 2, Fig. 1a); a 26.7% increase. At the village level, the prevalence fell in four villages, two of which had no infection and the other two only having one case of infection at follow-up. The prevalence increased in two villages: village 1 (6.4 *vs* 6.9%, *χ*^2^ = 0.0313, *df* = 1, *P* = 0.860) and village 5 (3.2 *vs* 9.0%, *χ*^2^ = 9.5139, *df* = 1, *P* = 0.002). Treatment coverage was low in village 1 (53.0%), but high in village 5 (95.4%) (Fig. 1a).

In Senegal, 1.8% (29/1613; 95% CI: 1.2–2.6%) of children aged 0–9 years-old were Amplicor-positive at baseline compared with 3.6% (64/1771; 95% CI: 2.8–4.6%) at follow-up (Table 2, Fig. 1b), representing a 100% increase. At the village level, the prevalence of Amplicor-positives stayed the same at 0% in four villages, fell in three villages, and increased in five (Fig. 1b).

### Change in active trachoma and ocular *C. trachomatis* infection for children present at both time-points

Approximately 80% of the village population was present at both time-points, for both countries (Table 3). In The Gambia, 847 censused children were present at both time-points. The immigration rate was 0.14, and the emigration rate was 0.18. In Senegal, 1282 censused children were present at both time-points. The immigration and emigration rates were 0.16 and 0.19, respectively.

**Table 3:** Clinical and Amplicor result status comparison between baseline and follow-up for children present at both time-points

	Active trachoma (TF and/or TI)	Amplicor-negative at both time-points	Amplicor-positive at both time-points	Amplicor-positive at baseline only	Amplicor-positive at follow-up only	Total
The Gambia	No active trachoma at either time-point	552	0	6	13	571
	Active trachoma at both time-points	68	6	1	7	82
	Active trachoma at baseline only	114	0	13	1	128
	Active trachoma at follow-up only	58	1	1	6	66
	Total	792	7	21	27	847
Senegal	No active trachoma at either time-point	995	0	4	14	1013
	Active trachoma at both time-points	38	5	1	9	53
	Active trachoma at baseline only	141	0	16	0	157
	Active trachoma at follow-up only	39	0	0	20	59
	Total	1213	5	21	43	1282

In The Gambia, of those who had active trachoma at baseline, 39.0% (82/210) had active trachoma at follow-up indicating that clinical signs resolved in 61.0% of children (Table 3). There was evidence of development of active trachoma between baseline and follow-up. Of all children present at both baseline and follow-up, 7.8% (66/847) developed active trachoma between baseline and follow-up, and 3.2% (27/847) became infected. Of those infected at any time-point, 14.5% (8/55) developed active trachoma between baseline and follow-up, whereas 7.3% (58/792) of children with no infection at either time-point developed active trachoma between baseline and follow-up. There was little evidence to suggest that those with infection at any time-point were significantly more likely to have developed active trachoma at follow-up than those without infection (*χ*^2^ = 3.7335, *df* = 1, *P* = 0.053). Of 28 children infected at baseline, 7 (25%) were also infected at follow-up. Of these, six were above the baseline median load of 405 *ompA* copies/swab: 4; 6317; 20,127; 62,131; 174,155; 185,246; and 318,918 *ompA* copies/swab (Table 4). 0.8% of 0 year-olds at follow-up (and therefore untreated) were Amplicor-positive.

**Table 4:** Median estimated organism load (*ompA* copies/swab) by clinical status of Amplicor-positive children

Clinical status	Gambian baseline			Gambian follow-up			Senegalese baseline			Senegalese follow-up		
	*n*	Median	Range	*n*	Median	Range	*n*	Median	Range	*n*	Median	Range
Normal	11	5	4–26	16	4	4–12,510	5	5	4–604	25	4	4–119,344
Active trachoma^a^	24	1556	4–3,008,063	29	8133	4–6,300,075	24	4670	4–174,507	39	13,260	4–564,558
Any TI	0	na	na	3	1252	4–1,899,670	4	48,558	3071–126,749	6	58,702	2920–564,558
Overall	35	405	4–3,002,063	45	22	4–6,300,075	29	2730	4–174,507	64	5855	4–564,558

^a^All those with active trachoma (TF and/or TI) had trachomatous inflammation-follicular (TF)

*Abbreviation*: na, not applicable

In Senegal, of those who had active trachoma at baseline, 25.2% (53/210) had active trachoma at follow-up, which indicates that clinical signs resolved in 74.8% of children by follow-up (Table 3). Of all children present at both baseline and follow-up, 4.6% (59/1282) developed active trachoma between baseline and follow-up, and 3.4% (43/1282) became Amplicor-positive. Of the 69 children with ocular *C. trachomatis* infection at any time-point, 29.0% (20/69) of children had developed active trachoma between baseline and follow-up. Of 1213 children not infected at either time-point, 39 (3.2%) developed active trachoma between baseline and follow-up. Those with infection at either time-point were significantly more likely to develop active trachoma between baseline and infection than those without infection (*χ*^2^ = 98.7551, *df* = 1, *P* < 0.001). Similar to the Gambian results, all but one of these were above the baseline median load of 2730 *ompA* copies/swab: 4268; 27,653; 46,823; 50,293; and 125,626 *ompA* copies/swab (Table 4). Of the 0 year-olds at follow-up, 4.0% were Amplicor-positive.

### Concordance between active trachoma (TF and/or TI) and ocular *C. trachomatis* infection

The difference between overall active trachoma and Amplicor-positive prevalence was significant in both countries at both baseline and follow-up (Gambia baseline: *χ*^2^ = 39.5518, *df* = 1, *P* < 0.001; Gambia follow-up: *χ*^2^ = 70.3365, *df* = 1, *P* < 0.001; Senegal baseline: *χ*^2^ = 107.4340, *df* = 1, *P* < 0.001; Senegal follow-up: *χ*^2^ = 252.1291, *df* = 1, *P* < 0.001). At baseline, only approximately 10% of children with active trachoma were Amplicor-positive in both countries (Table 2), whereas 68.6% (The Gambia) and 82.8% (Senegal) of Amplicor-positives had active trachoma. At follow-up, 14.0% and 27.5% of those with active trachoma were Amplicor-positive in The Gambia and Senegal, respectively. Approximately 60% of those who were Amplicor-positive had active trachoma in both countries (Table 2). The relationship between active trachoma and infection did not change between time-points, except for Senegal where there was evidence (*Z* = 2.1, *P* = 0.0364) that the decrease in proportion of those infected also having active trachoma was significantly lower at follow-up than at baseline in Senegal.

### One-year impact of MDA on organism load

For both countries and time-points, the organism load of Amplicor-positive children was lower for those who were clinically normal, than for those with active trachoma (Table 4). In The Gambia, the overall median organism load at baseline was 405 *ompA* copies/swab, compared with 22 copies/swab at follow-up (K-sample test: *P* = 1.000). However, median organism load increased between baseline (1556 copies/swab) and follow-up (8133 copies/swab) for those with active trachoma (K-sample test: *P* = 0.785). In Senegal, the median load was higher at follow-up than at baseline, both overall (5855 *vs* 2,730, *P* = 0.180) and for those with active trachoma (13,260 *vs* 4670; K-sample test: *P* = 0.439).

### One-year impact of MDA on circulating strains

Sequence was more likely to be recovered from samples with high *ompA* copy number estimates. Samples successfully sequenced for the five MLST regions contained a median of 44,952 *ompA* copies/swab, compared to 1142 *ompA* copies/swab where sequencing was incomplete or unsuccessful. Sequencing was not attempted where organism load was less than 30 *ompA* copies/swab or where there was no sample left. MLST sequencing was attempted on 99 (57.2%) of the 173 Amplicor-positive samples, resulting in 26 incomplete sequences and 73 complete sequences (Table 1). *OmpA* sequencing was attempted on 128 (74.0%) samples, successfully for 94 (73.4%) samples (Table 1). 72 (41.6%) Amplicor-positive samples were fully sequenced for MLST^6^ (the five MLST regions combined with *ompA*) (Table 1).

Samples from Senegal yielded exclusively genovar A sequences. In The Gambia, eight of 14 (57.1%) *ompA* sequences at baseline, and 13 of 18 (72.2%) of *ompA* sequences from follow-up samples, were genovar A with the rest genovar B.

**Fig. 2:**
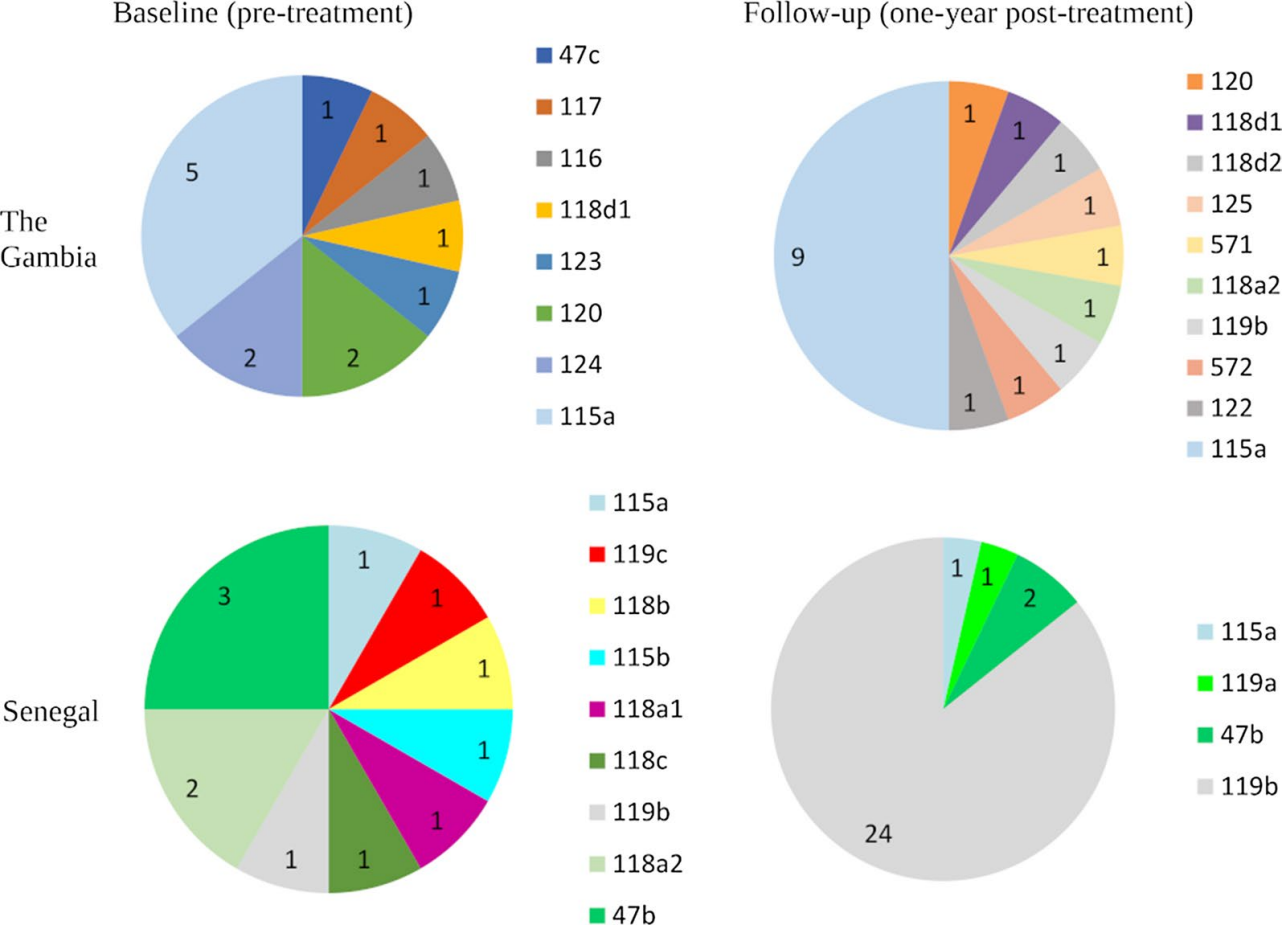
MLST^6^ variant frequency in The Gambia and Senegal before and one year after mass drug administration with azithromycin. MLST^6^ variants include *ompA* combined with all five MLST^5^ regions (*hctB*, CT058, CT144, CT172, *pbpB*)

**Fig. 3:**
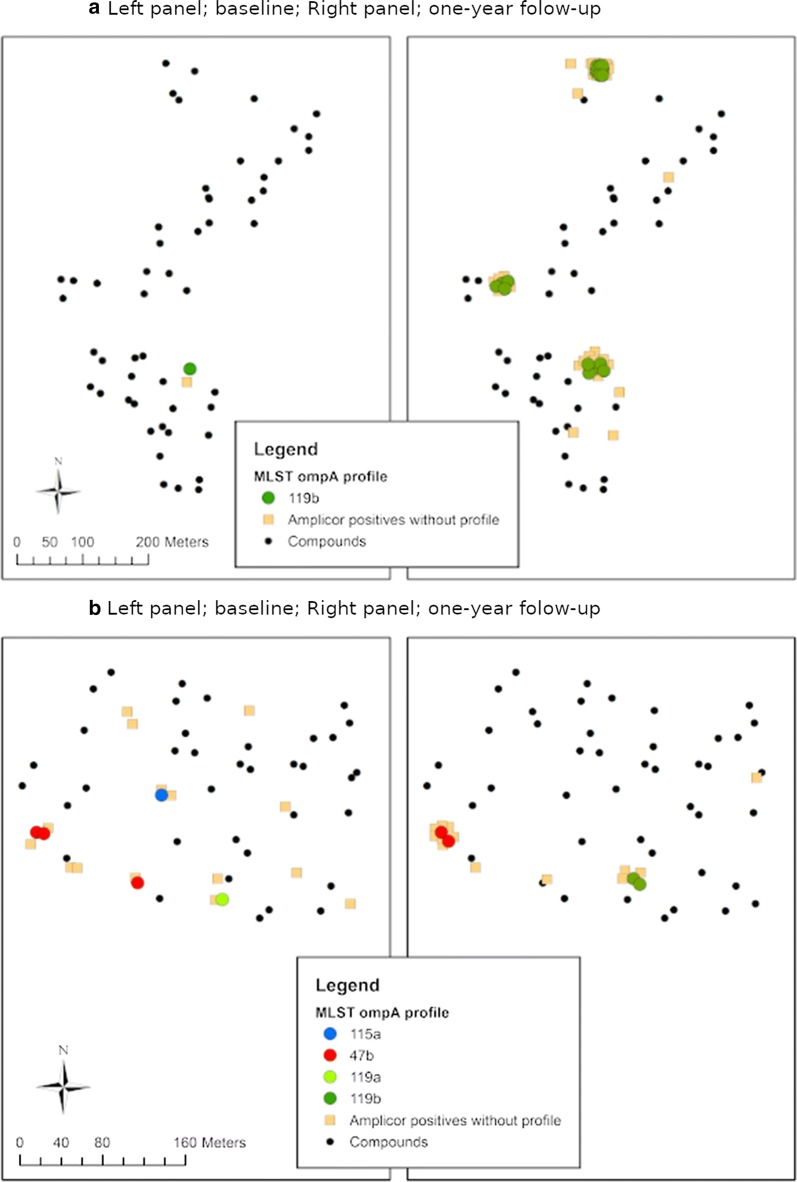
Geographical location of variants in Senegalese villages before and one year after mass drug administration with azithromycin. **a** Senegalese villages 6 and 11: “outbreak” of variant 119b at follow-up. **b** Senegalese village 3: variants 119b and 119a appeared at follow-up. Variant 47b remained in one household at follow-up, whereas the other variants disappeared between baseline and follow-up

**Fig. 4:**
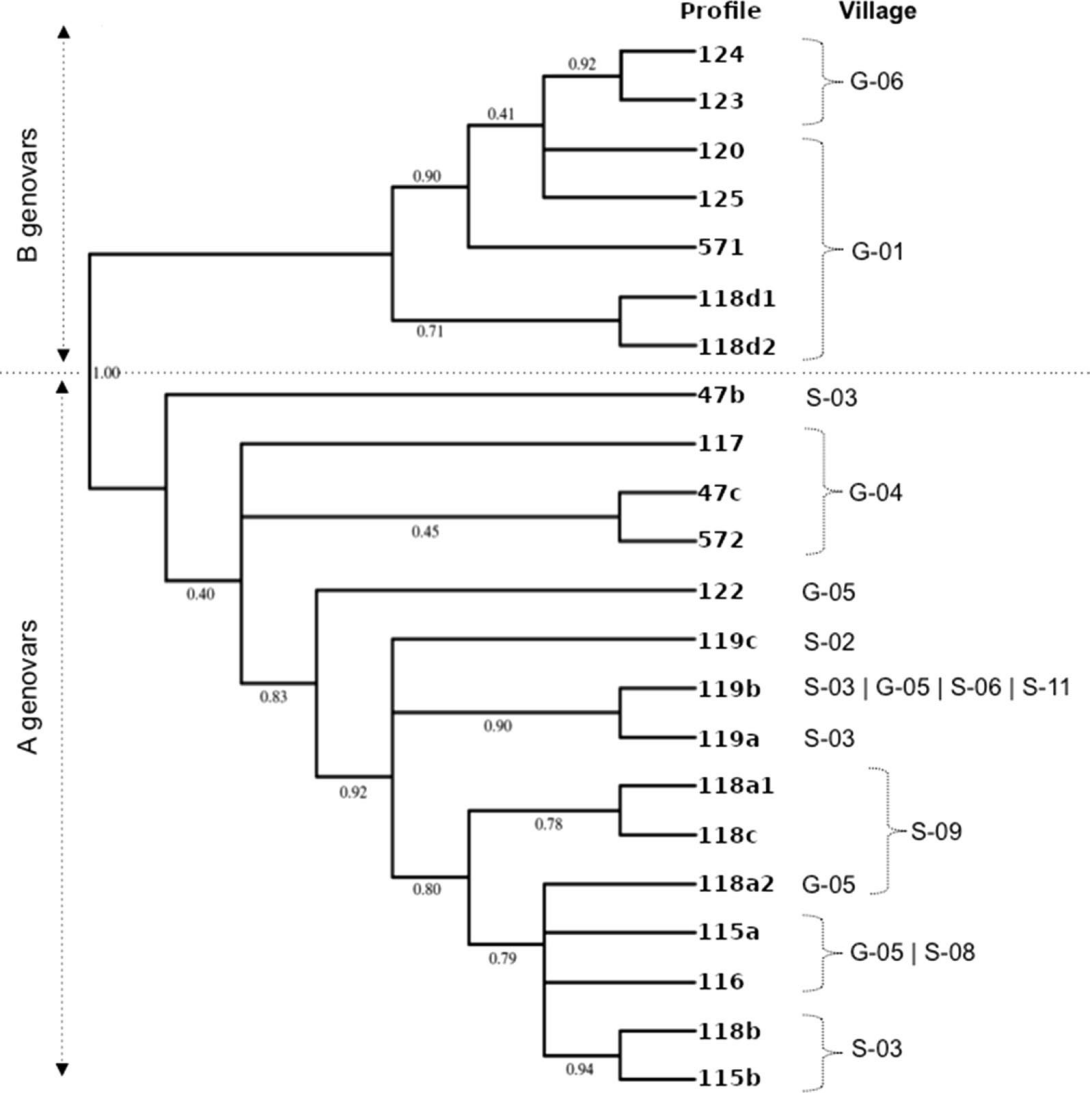
Phylogenetic relationships between the MLST^6^ variants. Bootstrap confidence values are shown at branch-points. Genotype: unique number of variant super-type; village: all villages have been coded. Senegalese villages are prefixed with an “S”, and Gambian villages prefixed with a “G”

For each of *ompA*, MLST and MLST^6^, both common and country-specific variants were present at both baseline and follow-up (Fig. 2). In Senegal, there was a decrease in the number of variants at follow-up, with one new variant appearing (119a) in Senegalese village S-03 (Fig. 3b). Variant 119a was closely related to 119b, which was also present at follow-up (Fig. 4). In Gambian village G-01, new variants 118d2, 125 and 571 joined variants 120 and 118d1 present at baseline. Figure 4 suggests that 125 is closely related to 120, 118d2 is closely related to 118d1, and 571 lies on its own branch. There was no evidence of a significant difference in variant frequency between baseline and follow-up in The Gambia (Fig. 2). In contrast, the frequency of *ompA*, *hctB*, CT144, and MLST^6^ variants differed significantly between baseline and follow-up in Senegal (Fisher’s exact test: *P* < 0.001). These differences are due to one variant (119b) that only featured once in 16 samples at baseline (8.3%), but accounted for 24 (85.7%) of the 28 samples at follow-up. This “outbreak” at follow-up is depicted in Fig. 3a. Mixed infections, which would have been indicated by mixed base calls in both directions of the sequencing, were not detected.

For both *ompA* (*n* = 94) and MLST^6^ (*n* = 72) types, in both countries and at both time-points, there was a significant over-representation of short genetic distances in sample pairs derived from the same, rather than different, villages, indicating that similar strains tended to cluster in villages (Table 5). Similarly, at household level, there was evidence of significant clustering of similar *ompA* and MLST^6^ types at follow-up in both countries.

**Table 5:** P-values for evidence of clustering at different cluster strata. Analyses based on comparisons of genetic distances between pairs of participants, based on the Hamming distance for pairs of participants in same or different cluster strata

Cluster strata	The Gambia				Senegal			
	*ompA*		MLST^6^		*ompA*		MLST^6^	
	Baseline	Follow-up	Baseline	Follow-up	Baseline	Follow-up	Baseline	Follow-up
Village	< 0.001	< 0.001	< 0.0001	< 0.0001	< 0.0001	< 0.0001	< 0.0001	< 0.0001
Household	0.156	< 0.002	0.37	0.03	0.229	< 0.0001	0.039	0.0029

*Note*: MLST^6^ variants include *ompA* combined with all five MLST^5^ regions (*hctB*, CT058, CT144, CT172, *pbpB*)

Simulation experiments suggested that at both time-points there was an over-representation of short genetic distances among sample pairs that were geographically close (e.g. < 5 km) (*P* < 0.001) and conversely that short geographical separations were over-represented among sample pairs that were genetically similar (e.g. to less than three base substitutions) (*P* < 0.01).

### Comparing MLST, complete *ompA* typing and WGS

**Fig. 5:**
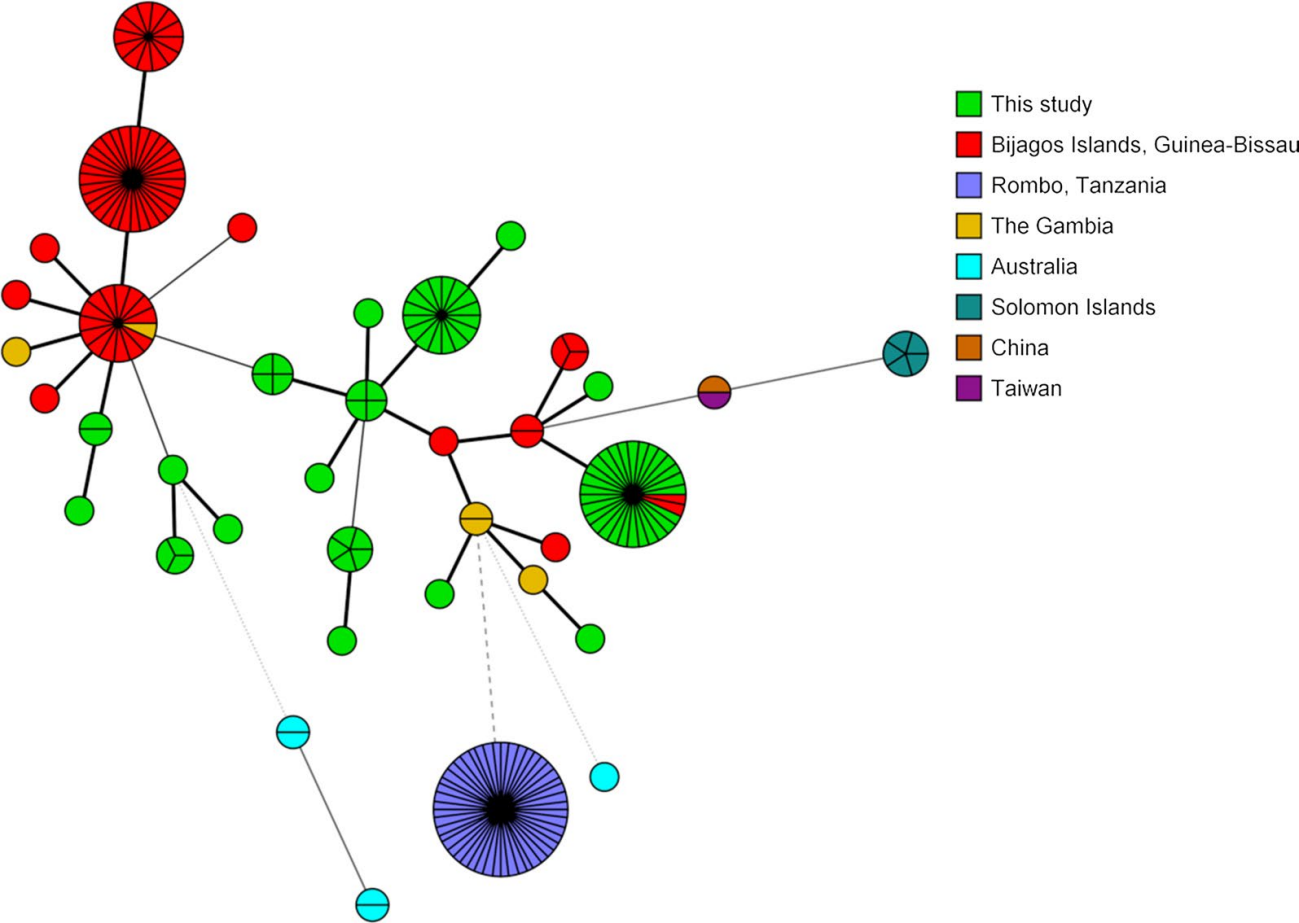
Minimum spanning tree analysis of publicly available ocular *C. trachomatis* isolates based on the six MLST target regions visualised by isolate origin. Sphere sizes indicate the numbers of samples in each sphere. Solid branches show single-locus variants, dashed branches show double-locus variants and dotted branches show triple-locus variants. All publicly available ocular *C. trachomatis* isolates were included in the analyses

In the sub-study comparing resolution achieved between WGS, *ompA* and MLST in a set of 71 WGS from Bijagos Islands, Guinea-Bissau [31], MLST discriminatory index was higher for the samples in this study compared with the Guinea-Bissau population (D = 0.825 *vs* D = 0.743), neither had discriminatory power considered to be necessary for molecular epidemiology (D = 0.95). Minimum spanning tree (MST) analyses showed minimal sharing of STs between these studies; however, STs from these studies did form overlapping clusters (Fig. 5). Equivalent sample size, MLST discriminatory power and clustering of STs support the population from Guinea-Bissau as a sensible comparator to this study.

**Fig. 6:**
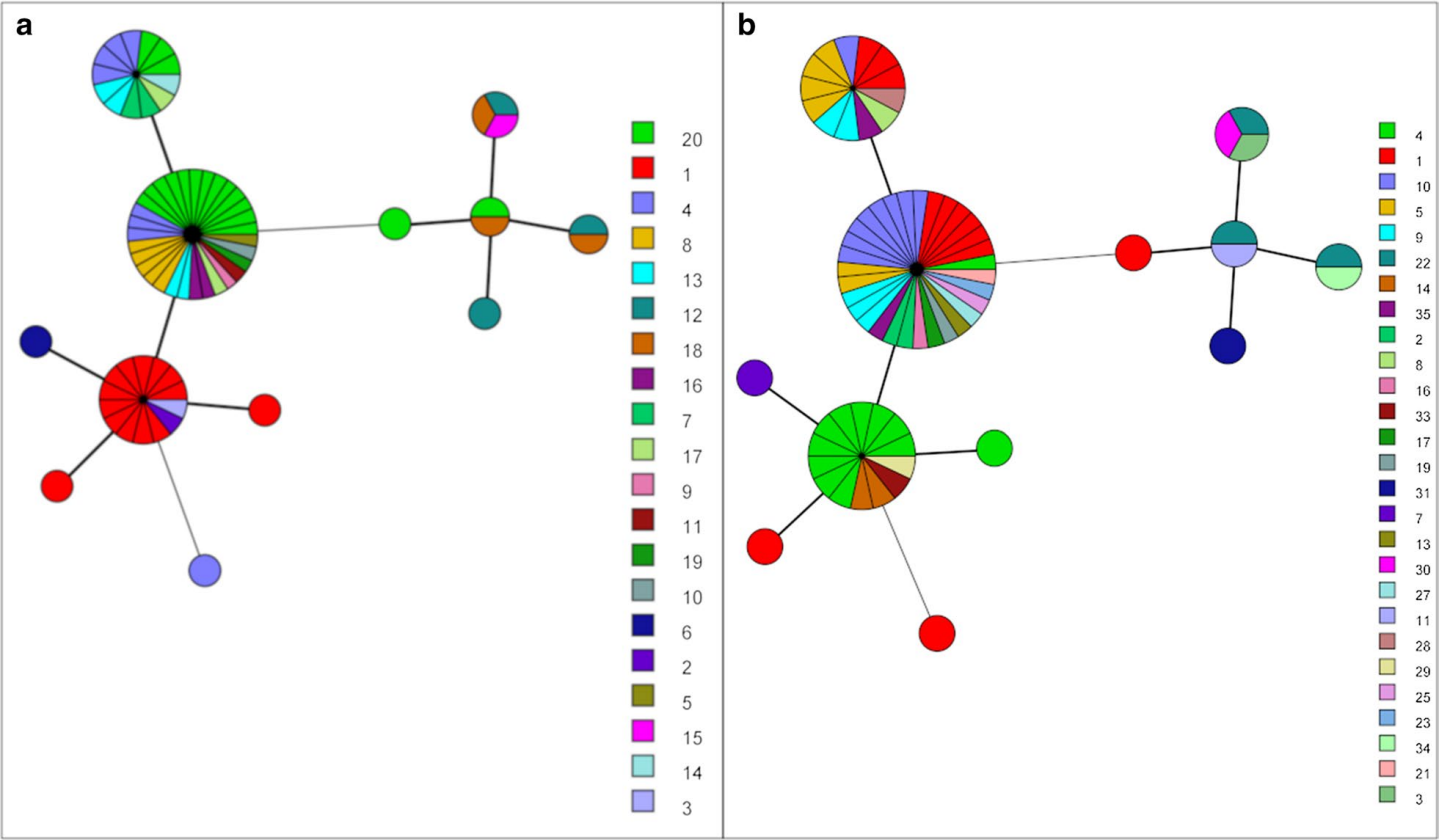
Minimum spanning tree analysis of ocular *C. trachomatis* isolates from the Bijagos Islands, Guinea-Bissau based on the six MLST target regions visualised by *ompA* type (**a**) and ‘WGS-type’ (**b**). Sphere sizes indicate the numbers of samples in each sphere. Solid branches show single-locus variants, dashed branches show double-locus variants and dotted branches show triple-locus variants. WGS with < 401 SNPs between them (fifth percentile of all pairwise differences) were defined as a ‘WGS-type’

MLST^6^, including partial typing of *ompA*, identified 12 STs in Guinea-Bissau (D = 0.743). Inference of full-length *ompA* from WGS identified 20 variants, with a discriminatory index of (D = 0.8805). All WGS were unique. Grouping WGS with < 401 SNPs between them (fifth percentile of all pairwise differences) identified 35 ‘WGS-types’, with a discriminatory index between MLST and *ompA* (D = 0.765). MST analyses of STs with isolates coloured by *ompA* type (Fig. 6a) or WGS-type (Fig. 6b) showed increased resolution using either method, and poor correlation with STs. Comparison of phylogenetic relationship between samples corroborated the disparity between WGS and STs (Additional file 1: Figure S1).

**Fig. 7:**
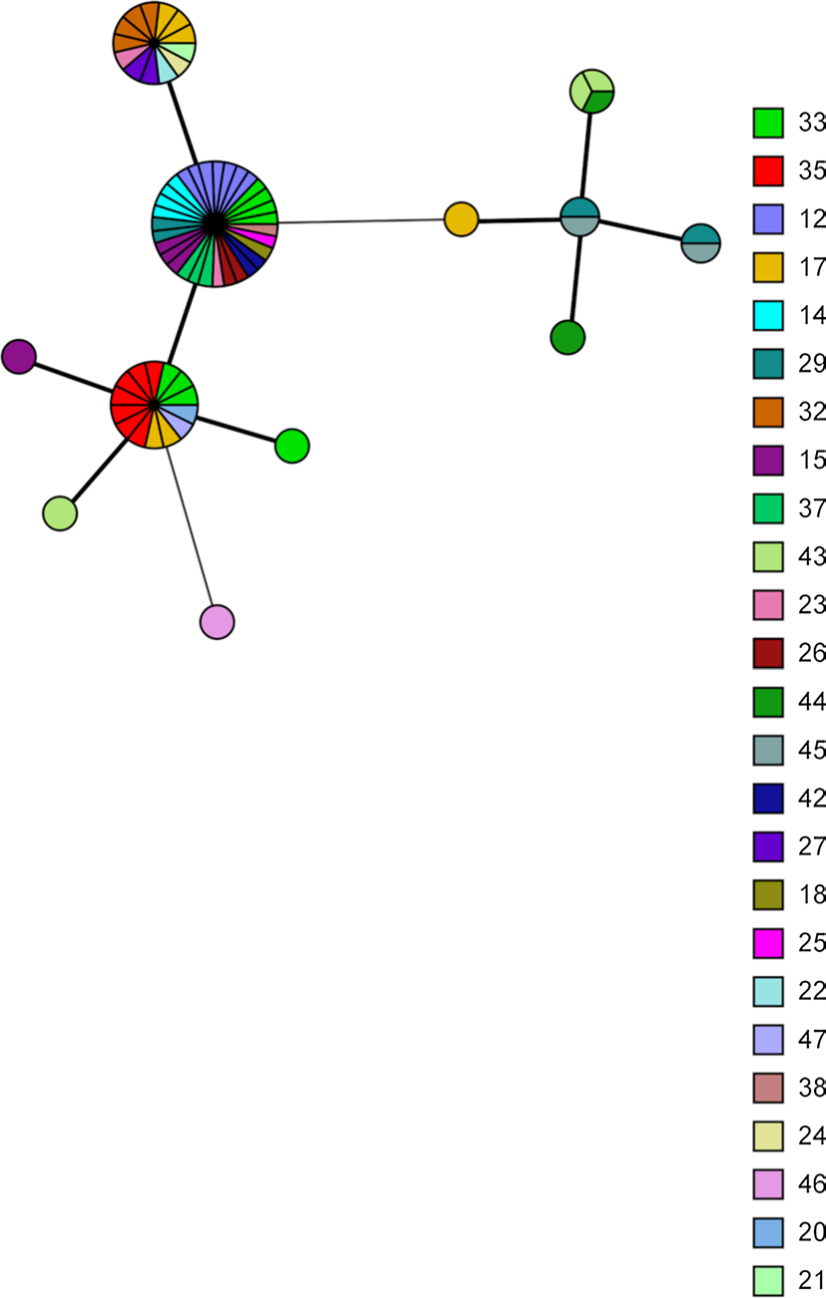
Minimum spanning tree analysis of ocular *C. trachomatis* isolates from the Bijagos Islands, Guinea-Bissau based on the six MLST target regions visualised by village of collection. Sphere sizes indicate the numbers of samples in each sphere. Solid branches show single-locus variants, dashed branches show double-locus variants and dotted branches show triple-locus variants

Phylogenetic analysis of WGS from Guinea-Bissau showed clustering by village of collection, supporting the utility of WGS for molecular epidemiology. MST analyses of STs with isolates coloured by village of collection showed evidence of clustering by village, however 6/12 STs were identified in multiple villages (Fig. 7).

## Discussion

In this study, we assessed the one-year impact of MDA with azithromycin in six Gambian and 12 Senegalese villages. Overall, active trachoma prevalence decreased in both countries. Conversely, there was no impact on ocular *C. trachomatis* infection, with an increase in prevalence observed in Senegal. There was a poor correlation between having active trachoma and being Amplicor-positive, whilst having higher organism load was associated with having active trachoma and more severe inflammation (TI). All Senegalese samples were genovar A, whereas The Gambia presented a mix of genovar A and B samples. MLST results demonstrated differences in number of circulating strains in Senegal but not in The Gambia, and also provided evidence of clustering at village and household levels.

There were some methodological differences between baseline and follow-up: at baseline, the Amplicor results came from the second-collected swab (the first-collected swab was processed by a POCT [25]), whereas at follow-up, Amplicor was performed on the first-collected swab. This could have affected the prevalence of infection observed at baseline, as the first-collected swab may have a higher load of infection than the second [43]. However, comparison between first- and second-collected swab results by others, and within this study (data not shown), has demonstrated excellent concordance [44, 45], unsurprising as the detection level of Amplicor is in the range of 1–10 elementary bodies [46, 47]. Another limitation is that the grader training did not follow the current globally standardised training system, which includes field-based inter-grader agreement (IGA) assessments because grading projected slides is not equivalent to field grading [48]. We did, however, attempt to validate grader examination. In The Gambia, a third grader (RLB) returned to some of the villages and verified with success the cases identified as clinically active. In Senegal, at the one-year follow-up, 50 sequential children were double-graded by both the Senegalese and Gambian ophthalmic nurses, once towards the beginning and once towards the end of the fieldwork, with each grader blind to the other’s diagnosis. For the first exercise, the kappa score was 0.8649, with an expected agreement of 70.40% and an observed agreement of 96.00%, showing almost perfect agreement. For the second exercise, the kappa score was 0.5524, with an expected agreement of 68.72% and an observed agreement of 86.00%, demonstrating moderate agreement, with the Senegalese grader tending to over-diagnose active trachoma compared with the Gambian grader. Furthermore, the graders knew that the villages had received MDA, which could have biased the graders to under-estimate the active trachoma prevalence. However, given the one-year follow-up results (all Gambian villages and four Senegalese villages with > 5% TF in 1–9 year-olds), combined with the grader validation exercises, any such bias seems to have had a minimal effect on overall results.

As no control villages were included in this study to compare what would have happened in the absence of MDA, the active trachoma prevalence declines observed cannot be attributed to MDA alone. The results could be due to random fluctuation, seasonal effects or regression to the mean of the villages as these communities were chosen to have a sufficiently high prevalence of disease to qualify them for MDA. Regression to the mean may reduce infection over time even without treatment [49]. Secular trend is another plausible explanation with studies showing that active trachoma can disappear in the absence of trachoma control programmes [50–53].

Our results question the reliance upon clinical signs to make decisions regarding implementing trachoma elimination measures. The WHO active trachoma indicator for programmatic decisions regarding MDA initiation and duration is the prevalence of TF in children aged 1–9 years. TF was a poor predictor for infection, but infection was a fairly reliable indicator for having TF, consistent with others’ findings in low prevalence or mass treated settings [7, 15, 44, 54–61]. Furthermore, our data indicate that the assertion by others that inclusion of TI would improve the association between clinical signs and infection [62] is likely context-specific. Of infected individuals, those with active trachoma (TF and/or TI) or “any TI” had a higher load of infection than those without disease. Furthermore, in Senegal, those with “any TI” had higher loads than those with active trachoma, demonstrating higher loads with increasing severity of inflammation, as previously noted in West Africa [7, 63]. Thus, high chlamydial load was a good marker for disease status, but active trachoma remained a poor predictor of infection. These data support the continued need for further work developing alternative indicators for diagnosing ocular *C. trachomatis* infection, as reliance on clinical signs is both poorly sensitive and specific [25, 64].

After one round of MDA, TF prevalence in 1–9 year-olds did not fall below the WHO 5% threshold for elimination of trachoma as a public health problem in any of The Gambia villages, and only in four of the Senegalese villages. This is expected, as the WHO recommendation is for three years of MDA before reassessment where TF prevalence is 10-29.9% [65]. Overall treatment coverage was above the WHO recommended minimum level of 80% [66] in The Gambia (data from Senegal were unreliable). However, at a village level, meeting the 80% target was not always consistent with favourable impact on active trachoma and infection prevalence. Despite others having demonstrated that a single MDA round with high coverage can be effective [13, 17], our results support the importance of implementation of the whole SAFE strategy, as high MDA coverage alone is not sufficient for reducing and sustaining trachoma prevalence to below the elimination threshold [12]. The lack of impact on infection indicates transmission continued to occur post-MDA. This indication of continued transmission is supported by the development of active trachoma and ocular *C. trachomatis* infection in children present at both baseline and follow-up in both countries, the appearance of new strains as determined by MLST at follow-up, and the observation that some children aged under one year at follow-up (and therefore untreated) had both evidence of infection and active trachoma. Of children infected at baseline still infected at follow-up, all but one had organism loads above the median at baseline, supporting others’ findings that those with high loads at baseline who receive treatment are more likely to be infected at follow-up [29, 63, 67]. We observed that approximately 20% of the population at follow-up was not present at baseline for both countries. Re-infection from migration (including into The Gambia from Senegal) has previously been reported [16, 68, 69]. This further highlights the importance of investing in the long-term “F” and “E” components of the SAFE strategy to limit transmission.

A proposed explanation for the success of MDA, despite the risk of outside re-infection, is the “Allee effect”, a concept taken from population biology [49]. It has been proposed that a variety of immunotypes allows *Chlamydia* to better evade the human immune response, and that if the effect of MDA was to reduce the diversity of chlamydial strains, the prevalence of infection may not be able to return to previous levels [49]. The difference in the effect of MDA between the communities may therefore be due to the variety of immunotypes circulating. The MLST system enabled us to identify a number of different circulating strains in both countries, at both time-points. In Senegal, we observed a reduction in strain diversity following MDA, similar to that observed with *ompA* types in The Gambia previously [21]. The reduction in diversity in Senegal only may possibly reflect the simultaneous MDA of the district compared to the treatment of isolated villages independently of their surrounding settlements in The Gambia, which may have increased vulnerability to re-infection. The reduction in diversity in Senegal, is however inconsistent with the Allee effect hypothesis, since an increase in overall ocular *C. trachomatis* prevalence was observed at follow-up.

Different typing schemes have been developed and implemented, and have been interpreted as providing evidence of intra-familial transmission of trachoma [21, 54, 70–72], and of clustering of related trachoma infections at the household level [73, 74]. *OmpA* genotyping has also suggested that some individuals may be persistently infected with the same strain [70, 71, 75], and that recombination and mixed infections both occur [76–78]. In genital *C. trachomatis* infections, MLST has a considerably higher discriminatory capacity than *ompA* typing [79, 80] and was therefore used in this study to investigate its usefulness in trachoma surveillance. We additionally evaluated the relationship between MLST and whole-genome sequence variation as a sub-study, using a population from Bijagos Islands, Guinea-Bissau.

MLST provided evidence that some individuals infected at both time-points were re-infected rather than persistently infected, and suggested that a single re-infecting strain (119b) had spread widely in a Senegalese village. We also noted geographical clustering of genetically similar strains, which is reassuring in that it is consistent with the generally accepted notion that most individuals acquire ocular *C. trachomatis* infection from members of the same household or community [63, 67]. However, almost 60% of the ocular infections we found could not be fully typed, restricting the amount of data for comparisons. The types that we successfully determined are biased towards the samples containing more *ompA* copies. This, independently of the discriminatory ability of the typing itself, further reduces its utility for molecular epidemiology and precludes the analysis of some key questions such as whether strains vary in their transmissibility or in their tendency to cause ocular inflammation or disease sequelae. We previously reported distinct strain types associated with variation in sample *ompA* copy number [21], but if that had been the case in this study, we would not have detected it. The typing scheme proved quite demanding of sample, with some samples being used up entirely. These problems may be overcome in future by the development and incorporation of array-based typing methods into the MLST^6^ scheme [81].

The sub-study comparing WGS and MLST in a population from Bijagos Islands, Guinea-Bissau highlighted the improved resolution achieved with WGS. MST and phylogenetic analyses also identified considerable discrepancy in the relatedness of isolates between WGS and MLST. WGS of *C. trachomatis* is possible directly from clinical samples with as few as 500 genome copies [31, 82–84]. However, it is still relatively expensive compared to MLST. Applying Simpson’s discriminatory index to WGS produced a value of zero. Even attempts to define ‘WGS-types’ produced a lower discriminatory index than *ompA* typing. This is due to the high prevalence of unique isolates and ‘WGS-types’, respectively, which are discounted when calculating the index. MLST, including partial *ompA* typing, provided greater discriminatory power than complete *ompA* typing in this study. The opposite was true for inferred MLST and *ompA* types from Guinea-Bissau. The inconsistency of the low discriminatory power of MLST in ocular *C. trachomatis* and the increased resolution of WGS, suggests that novel targets are required if MLST is to be utilised in studies of trachoma.

In addition to overcoming these methodological challenges of MLST, to better understand the impact of MDA on active trachoma and ocular *C. trachomatis* infection, it would be helpful to have longer follow-up and the inclusion of control villages. Additional information, such as on travel patterns in both The Gambia and Senegal, could help understand the contribution of imported infection on long-term active trachoma and infection prevalence, circulating strains, and ultimately the success of trachoma elimination programme interventions.

## Conclusions

We found that one round of MDA with azithromycin led to an overall decline in active trachoma prevalence but no impact on ocular *C. trachomatis* infection, with heterogeneity between the villages studied. The poor correlation between active trachoma and infection prevalence supports the need for further work on alternative indicators to clinical signs for diagnosing ocular *C. trachomatis* infection. The use of MLST typing has potential molecular epidemiology utility, including better understanding of transmission dynamics, although relationship to whole-genome sequence variability requires further exploration.

## Supplementary information

**Additional file 1: Figure S1.** Maximum likelihood reconstruction of whole genome and MLST phylogeny of ocular *C. trachomatis* sequences from the Bijagos Islands, Guinea-Bissau. MLST sequences were concatenated to create a complete MLST sequence per individual. Multiple MLST and genome alignments were generated using progressiveMauve. Phylogenies were computed using RaxML [5] and visualised in R. MLST and WGS phylogenies were compared using R package *dendextend* [6]. Isolates which were separated by < 90% of bootstrap replicates, using MLST and WGS respectively, are highlighted in the same colour. The scale-bar indicates evolutionary distance.

## Data Availability

Data supporting the conclusions of this article are included within the article. The datasets used and/or analysed during the present study are available from the corresponding author upon reasonable request.

## Abbreviations

CI: confidence interval
GPS: global positioning system
IGA: inter-grader agreement
ITI: International Trachoma Initiative
MDA: mass drug administration
MLST: multi-locus sequence typing
MST: minimum spanning tree
NECP: National Eye Care Programme
PCR: polymerase chain reaction
POCT: point-of-care test
SAFE: Surgery, Antibiotics, Facial cleanliness, Environmental improvement
ST: sequence type
TEO: tetracycline eye ointment
TF: trachomatous inflammation-follicular
TI: trachomatous inflammation-intense
WGS: whole-genome sequence
WHO: World Health Organization

## References

[1] WHO. WHO alliance for the global elimination of trachoma by 2020: progress report on elimination of trachoma, 2017. Wkly Epidemiol Rec. 2018;93:369–80. https://apps.who.int/iris/bitstream/handle/10665/272967/WER9326.pdf?ua=1. Accessed 23 Apr 2019.

[2] WHO. Planning meeting for the global elimination of trachoma, Geneva, 25–28 November 1996. Geneva: World Health Organization; 1997. http://whqlibdoc.who.int/hq/1997/WHO_PBL_97.60.pdf. Accessed 23 Apr 2019.

[3] WHO. Technical consultation on trachoma surveillance, September 11–12, 2014. Technical Advisory Group on Neglected Tropical Diseases. Task Force for Global Health, Decatur, USA. http://apps.who.int/iris/bitstream/10665/174085/1/WHO_HTM_NTD_2015.02_en.pdf2015. Accessed 23 Apr 2019.

[4] Dolin PJ, Faal H, Johnson GJ, Ajewole J, Mohamed AA, Lee PS (1998). Trachoma in The Gambia. Br J Ophthalmol.

[5] Harding-Esch EM, Edwards T, Sillah A, Sarr I, Roberts CH, Snell P (2009). Active trachoma and ocular *Chlamydia trachomatis* infection in two Gambian regions: on course for elimination by 2020?. PLoS Negl Trop Dis.

[6] Harding-Esch EM, Sillah A, Edwards T, Burr SE, Hart JD, Joof H (2013). Mass treatment with azithromycin for trachoma: when is one round enough? Results from the PRET Trial in the Gambia. PLoS Negl Trop Dis.

[7] Burton MJ, Holland MJ, Faal N, Aryee EA, Alexander ND, Bah M (2003). Which members of a community need antibiotics to control trachoma? Conjunctival *Chlamydia trachomatis* infection load in Gambian villages. Invest Ophthalmol Vis Sci.

[8] Saal MB, Schemann JF, Saar B, Faye M, Momo G, Mariotti S (2003). Trachoma in Senegal: results of a national survey. Med Trop.

[9] Faye M, Kuper H, Dineen B, Bailey R (2006). Rapid assessment for prioritisation of trachoma control at community level in one district of the Kaolack Region, Senegal. Trans R Soc Trop Med Hyg.

[10] Evans JR, Solomon AW, Jumar R, Perez A, Singh BP, Srivastava RM (2019). Antibiotics for trachoma. Cochrane Database Syst Rev.

[11] Bobba S, Phelan S, Schierhout G, McManus H, Menzies R, Kaldor J (2018). A systemic review of community level interventions in reducing the prevalence of active trachoma. Clin Exp Ophthalmol.

[12] Emerson PM, Hooper PJ, Sarah V (2017). Progress and projections in the program to eliminate trachoma. PLoS Negl Trop Dis.

[13] Solomon AW, Holland MJ, Alexander ND, Massae PA, Aguirre A, Natividad-Sancho A (2004). Mass treatment with single-dose azithromycin for trachoma. N Engl J Med.

[14] West SK, Munoz B, Mkocha H, Gaydos C, Quinn T (2007). Trachoma and ocular *Chlamydia trachomatis* were not eliminated three years after two rounds of mass treatment in a trachoma hyperendemic village. Invest Ophthalmol Vis Sci.

[15] Schachter J, West SK, Mabey D, Dawson CR, Bobo L, Bailey R (1999). Azithromycin in control of trachoma. Lancet.

[16] Burton MJ, Holland MJ, Makalo P, Aryee EA, Alexander ND, Sillah A (2005). Re-emergence of *Chlamydia trachomatis* infection after mass antibiotic treatment of a trachoma-endemic Gambian community: a longitudinal study. Lancet.

[17] Solomon AW, Harding-Esch E, Alexander ND, Aguirre A, Holland MJ, Bailey RL (2008). Two doses of azithromycin to eliminate trachoma in a Tanzanian community. N Engl J Med.

[18] Lakew T, House J, Hong KC, Yi E, Alemayehu W, Melese M (2009). Reduction and return of infectious trachoma in severely affected communities in Ethiopia. PLoS Negl Trop Dis.

[19] Keenan JD, Lakew T, Alemayehu W, Melese M, Porco TC, Yi E (2010). Clinical activity and polymerase chain reaction evidence of chlamydial infection after repeated mass antibiotic treatments for trachoma. Am J Trop Med Hyg.

[20] Keenan JD, Tadesse Z, Gebresillasie S, Shiferaw A, Zerihun M, Emerson PM (2018). Mass azithromycin distribution for hyperendemic trachoma following a cluster-randomized trial: a continuation study of randomly reassigned subclusters (TANA II). PLoS Med.

[21] Andreasen AA, Burton MJ, Holland MJ, Polley S, Faal N, Mabey DC (2008). *Chlamydia trachomatis* ompA variants in trachoma: what do they tell us?. PLoS Negl Trop Dis.

[22] Klint M, Fuxelius HH, Goldkuhl RR, Skarin H, Rutemark C, Andersson SG (2007). High-resolution genotyping of *Chlamydia trachomatis* strains by multilocus sequence analysis. J Clin Microbiol.

[23] Jurstrand M, Christerson L, Klint M, Fredlund H, Unemo M, Herrmann B (2010). Characterisation of *Chlamydia trachomatis* by ompA sequencing and multilocus sequence typing in a Swedish county before and after identification of the new variant. Sex Transm Infect.

[24] Fredlund H, Falk L, Jurstrand M, Unemo M (2004). Molecular genetic methods for diagnosis and characterisation of *Chlamydia trachomatis* and *Neisseria gonorrhoeae*: impact on epidemiological surveillance and interventions. APMIS.

[25] Harding-Esch E, Holland MJ, Schemann JF, Molina S, Sarr I, Andreasen AR (2011). Diagnostic accuracy of a prototype point-of-care test for ocular *Chlamydia trachomatis* under field conditions in The Gambia and Senegal. PLoS Negl Trop Dis.

[26] WHO. Report of the eighth meeting of the WHO alliance for the global elimination of blinding trachoma, Geneva: World Health Organization; 2004. http://www.who.int/blindness/publications/GET_8_Report.pdf2004. Accessed 23 Apr 2019.

[27] Thylefors B, Dawson CR, Jones BR, West SK, Taylor HR (1987). A simple system for the assessment of trachoma and its complications. Bull World Health Organ.

[28] Landis JR, Koch GG (1977). The measurement of observer agreement for categorical data. Biometrics.

[29] Solomon AW, Holland MJ, Burton MJ, West SK, Alexander ND, Aguirre A (2003). Strategies for control of trachoma: observational study with quantitative PCR. Lancet.

[30] Herrmann B, Isaksson J, Ryberg M, Tangrot J, Saleh I, Versteeg B (2015). Global multilocus sequence type analysis of *Chlamydia trachomatis* strains from 16 countries. J Clin Microbiol.

[31] Last AR, Pickering H, Roberts CH, Coll F, Phelan J, Burr SE (2018). Population-based analysis of ocular *Chlamydia trachomatis* in trachoma-endemic West African communities identifies genomic markers of disease severity. Genome Med.

[32] Edgar RC (2004). MUSCLE: multiple sequence alignment with high accuracy and high throughput. Nucleic Acids Res.

[33] Guindon S, Gascuel O (2003). A simple, fast, and accurate algorithm to estimate large phylogenies by maximum likelihood. Syst Biol.

[34] R Development Core Team. R: a language and environment for statistical computing. Vienna: R Foundation for Statistical Computing; 2016. https://www.R-project.org/. Accessed 23 Apr 2019.

[35] Hunter PR, Gaston MA (1988). Numerical index of the discriminatory ability of typing systems: an application of Simpson’s index of diversity. J Clin Microbiol.

[36] van Belkum A, Tassios PT, Dijkshoorn L, Haeggman S, Cookson B, Fry NK (2007). Guidelines for the validation and application of typing methods for use in bacterial epidemiology. Clin Microbiol Infect.

[37] Gupta A, Jordan IK, Rishishwar L (2017). stringMLST: a fast k-mer based tool for multilocus sequence typing. Bioinformatics.

[38] Versteeg B, Bruisten SM, van der Ende A, Pannekoek Y (2016). Does typing of *Chlamydia trachomatis* using housekeeping multilocus sequence typing reveal different sexual networks among heterosexuals and men who have sex with men?. BMC Infect Dis.

[39] Langmead B, Salzberg SL (2012). Fast gapped-read alignment with Bowtie 2. Nat Methods.

[40] Li H, Handsaker B, Wysoker A, Fennell T, Ruan J, Homer N (2009). The sequence alignment/map format and SAMtools. Bioinformatics.

[41] Stamatakis A (2014). RAxML version 8: a tool for phylogenetic analysis and post-analysis of large phylogenies. Bioinformatics.

[42] Galili T (2015). dendextend: an R package for visualizing, adjusting and comparing trees of hierarchical clustering. Bioinformatics.

[43] Michel CE, Solomon AW, Magbanua JP, Massae PA, Huang L, Mosha J (2006). Field evaluation of a rapid point-of-care assay for targeting antibiotic treatment for trachoma control: a comparative study. Lancet.

[44] Miller K, Schmidt G, Melese M, Alemayehu W, Yi E, Cevallos V (2004). How reliable is the clinical exam in detecting ocular chlamydial infection?. Ophthalmic Epidemiol.

[45] Chidambaram JD, Alemayehu W, Melese M, Lakew T, Yi E, House J (2006). Effect of a single mass antibiotic distribution on the prevalence of infectious trachoma. JAMA.

[46] Miyashita N, Lijima Y, Matsumoto A (1994). Evaluation of the sensitivity and specificity of polymerase chain reaction test kit, AMPLICOR *Chlamydia trachomatis*. Microbiol Immunol.

[47] Shattock RM, Patrizio C, Simmonds P, Sutherland S (1998). Detection of *Chlamydia trachomatis* in genital swabs: comparison of commercial and in house amplification methods with culture. Sex Transm Infect.

[48] Solomon AW, Pavluck AL, Courtright P, Aboe A, Adamu L, Alemayehu W (2015). The global trachoma mapping project: methodology of a 34-country population-based study. Ophthalmic Epidemiol..

[49] Chidambaram JD, Lee DC, Porco TC, Lietman TM (2005). Mass antibiotics for trachoma and the Allee effect. Lancet Infect Dis.

[50] Dolin PJ, Faal H, Johnson GJ, Minassian D, Sowa S, Day S (1997). Reduction of trachoma in a sub-Saharan village in absence of a disease control programme. Lancet.

[51] Numazaki K, Ikehata M, Chiba S, Aoki K (1997). Reduction of trachoma in absence of a disease-control programme. Lancet.

[52] Hoechsmann A, Metcalfe N, Kanjaloti S, Godia H, Mtambo O, Chipeta T (2001). Reduction of trachoma in the absence of antibiotic treatment: evidence from a population-based survey in Malawi. Ophthalmic Epidemiol.

[53] Jha H, Chaudary JS, Bhatta R, Miao Y, Osaki-Holm S, Gaynor B (2002). Disappearance of trachoma from western Nepal. Clin Infect Dis.

[54] Bailey RL, Hampton TJ, Hayes LJ, Ward ME, Whittle HC, Mabey DC (1994). Polymerase chain reaction for the detection of ocular chlamydial infection in trachoma-endemic communities J Infect Dis.

[55] Baral K, Osaki S, Shreshta B, Panta CR, Boulter A, Pang F (1999). Reliability of clinical diagnosis in identifying infectious trachoma in a low-prevalence area of Nepal. Bull World Health Organ.

[56] Thein J, Zhao P, Liu H, Xu J, Jha H, Miao Y (2002). Does clinical diagnosis indicate ocular chlamydial infection in areas with a low prevalence of trachoma?. Ophthalmic Epidemiol.

[57] Solomon AW, Peeling RW, Foster A, Mabey DC (2004). Diagnosis and assessment of trachoma. Clin Microbiol Rev.

[58] Cumberland P, Edwards T, Hailu G, Harding-Esch E, Andreasen A, Mabey D (2008). The impact of community level treatment and preventative interventions on trachoma prevalence in rural Ethiopia. Int J Epidemiol.

[59] Ngondi J, Gebre T, Shargie EB, Adamu L, Ejigsemahu Y, Teferi T (2009). Evaluation of three years of the SAFE strategy (Surgery, Antibiotics, Facial cleanliness and Environmental improvement) for trachoma control in five districts of Ethiopia hyperendemic for trachoma. Trans R Soc Trop Med Hyg.

[60] Ramadhani AM, Derrick T, Macleod D, Holland MJ, Burton MJ (2016). The relationship between active trachoma and ocular *Chlamydia trachomatis* infection before and after mass antibiotic treatment. PLoS Negl Trop Dis.

[61] Amza A, Kadri B, Nassirou B, Cotter SY, Stoller NE, West SK (2019). Community-level association between clinical trachoma and ocular chlamydia infection after mass azithromycin distribution in a mesoendemic region of Niger. Ophthalmic Epidemiol.

[62] Zambrano AI, Munoz BE, Mkocha H, Dize L, Gaydos CA, Quinn T (2017). Measuring trachomatous inflammation-intense (TI) when prevalence is low provides data on infection with *Chlamydia trachomatis*. Invest Ophthalmol Vis Sci.

[63] Last AR, Burr SE, Harding-Esch E, Cassama E, Nabicassa M, Roberts CH (2017). The impact of a single round of community mass treatment with azithromycin on disease severity and ocular *Chlamydia trachomatis* load in treatment-naive trachoma-endemic island communities in West Africa. Parasit Vectors.

[64] WHO. World Health Organization strategic and technical advisory group for neglected tropical diseases. Working group on monitoring and evaluation. Trachoma alternative indicators study data review, 31 August–1 September 2016. Geneva: World Health Organization; 2017. http://apps.who.int/iris/bitstream/10665/259397/1/WHO-HTM-NTD-PCT-2017.10-eng.pdf?ua=12016. Accessed 23 Apr 2019.

[65] WHO. Report of the third global scientific meeting on trachoma, Baltimore, 19–20 July 2010. Geneva: World Health Organization; 2010. http://www.who.int/blindness/publications/WORLDHEALTHORGANIZATIONGSMmtgreportFINALVERSION.pdf?ua=1. Accessed 23 Apr 2019.

[66] WHO (2006). Trachoma control—a guide for programme managers.

[67] West SK, Munoz B, Mkocha H, Holland MJ, Aguirre A, Solomon AW (2005). Infection with *Chlamydia trachomatis* after mass treatment of a trachoma hyperendemic community in Tanzania: a longitudinal study. Lancet.

[68] Shah NA, House J, Lakew T, Alemayehu W, Halfpenny C, Hong KC (2010). Travel and implications for the elimination of trachoma in Ethiopia. Ophthalmic Epidemiol.

[69] Burton M, Holland MJ, Makalo P, Aryee E, Sillah A, Cohert S (2009). Controlling hypoendemic trachoma in The Gambia with a single mass antibiotic treatment: a five-year longitudinal study.

[70] Bobo LD, Novak N, Munoz B, Hsieh YH, Quinn TC, West S (1997). Severe disease in children with trachoma is associated with persistent *Chlamydia trachomatis* infection. J Infect Dis.

[71] Smith A, Munoz B, Hsieh YH, Bobo L, Mkocha H, West S (2001). OmpA genotypic evidence for persistent ocular *Chlamydia trachomatis* infection in Tanzanian village women. Ophthalmic Epidemiol.

[72] Taylor HR, Rapoza PA, West S, Johnson S, Munoz B, Katala S (1989). The epidemiology of infection in trachoma. Invest Ophthalmol Vis Sci.

[73] Bailey R, Hayes L, Pickett M, Whittle H, Ward M, Mabey D (1994). Molecular epidemiology of trachoma in a Gambian village. Br J Ophthalmol.

[74] Bain DL, Lietman T, Rasmussen S, Kalman S, Fan J, Lammel C (2001). Chlamydial genovar distribution after community wide antibiotic treatment. J Infect Dis.

[75] Hsieh YH, Bobo LD, Quinn TC, West SK (2001). Determinants of trachoma endemicity using *Chlamydia trachomatis* ompA DNA sequencing. Microbes Infect.

[76] Millman KL, Tavare S, Dean D (2001). Recombination in the ompA gene but not the omcB gene of *Chlamydia* contributes to serovar-specific differences in tissue tropism, immune surveillance, and persistence of the organism. J Bacteriol.

[77] Brunelle BW, Sensabaugh GF (2006). The ompA gene in *Chlamydia trachomatis* differs in phylogeny and rate of evolution from other regions of the genome. Infect Immun.

[78] Hayes LJ, Bailey RL, Mabey DC, Clarke IN, Pickett MA, Watt PJ (1992). Genotyping of *Chlamydia trachomatis* from a trachoma-endemic village in the Gambia by a nested polymerase chain reaction: identification of strain variants. J Infect Dis.

[79] Bom RJ, Christerson L, Schim van der Loeff MF, Coutinho RA, Herrmann B, Bruisten SM (2011). Evaluation of high-resolution typing methods for *Chlamydia trachomatis* in samples from heterosexual couples. J Clin Microbiol.

[80] Gravningen K, Christerson L, Furberg AS, Simonsen GS, Odman K, Stahlsten A (2012). Multilocus sequence typing of genital *Chlamydia trachomatis* in Norway reveals multiple new sequence types and a large genetic diversity. PLoS ONE.

[81] Christerson L, Ruettger A, Gravningen K, Ehricht R, Sachse K, Herrmann B (2011). High-resolution genotyping of *Chlamydia trachomatis* by use of a novel multilocus typing DNA microarray. J Clin Microbiol.

[82] Seth-Smith HM, Harris SR, Skilton RJ, Radebe FM, Golparian D, Shipitsyna E (2013). Whole-genome sequences of *Chlamydia trachomatis* directly from clinical samples without culture. Genome Res.

[83] Christiansen MT, Brown AC, Kundu S, Tutill HJ, Williams R, Brown JR (2014). Whole-genome enrichment and sequencing of *Chlamydia trachomatis* directly from clinical samples. BMC Infect Dis.

[84] Butcher RM, Sokana O, Jack K, Macleod CK, Marks ME, Kalae E (2016). Low prevalence of conjunctival infection with *Chlamydia trachomatis* in a treatment-naive trachoma-endemic region of the Solomon Islands. PLoS Negl Trop Dis.

