# Supplementary figures and images for: Impact of a single round of mass drug administration with azithromycin on active trachoma and ocular Chlamydia trachomatis prevalence and circulating strains in The Gambia and Senegal
Source: Parasit Vectors. 2019 Oct 22;12:497. doi: 10.1186/s13071-019-3743-x (PMC6805539; doi:10.1186/s13071-019-3743-x)

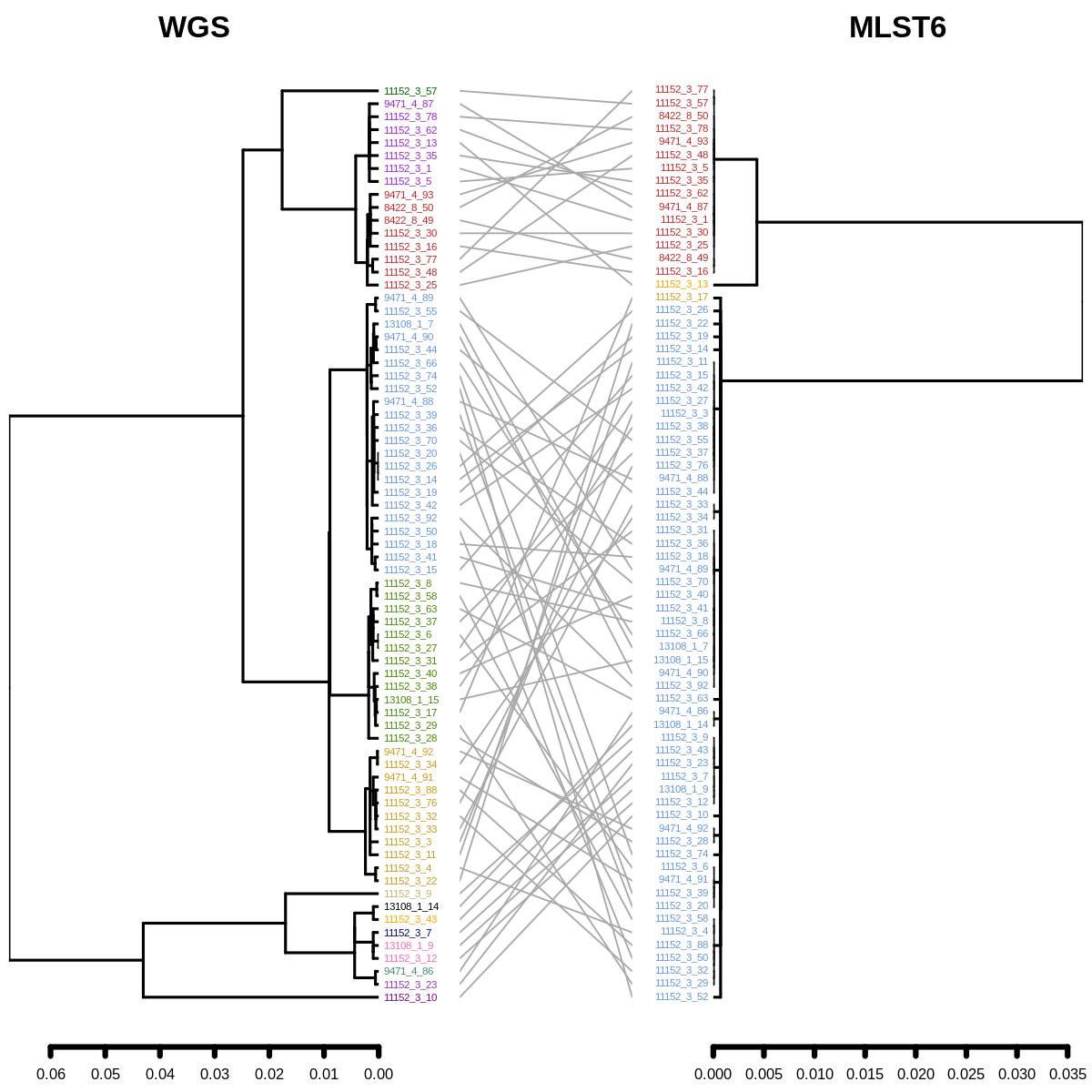

Supplement: Supplementary file 1 — Additional file 1: Figure S1. Maximum likelihood reconstruction of whole genome and MLST phylogeny of ocular C. trachomatis sequences from the Bijagos Islands, Guinea-Bissau. MLST sequences were concatenated to create a complete MLST sequence per individual. Multiple MLST and genome alignments were generated using progressiveMauve. Phylogenies were computed using RaxML [5] and visualised in R. MLST and WGS phylogenies were compared using R package dendextend [6]. Isolates which were separated by < 90% of bootstrap replicates, using MLST and WGS respectively, are highlighted in the same colour. The scale-bar indicates evolutionary distance. [file 13071_2019_3743_MOESM1_ESM.tiff]
